# Correlation of Glucosinolates and Volatile Constituents of Six Brassicaceae Seeds with Their Antioxidant Activities Based on Partial Least Squares Regression

**DOI:** 10.3390/plants11091116

**Published:** 2022-04-20

**Authors:** Noha Khalil, Haidy A. Gad, Nawal M. Al Musayeib, Mokhtar Bishr, Mohamed L. Ashour

**Affiliations:** 1 Department of Pharmacognosy and Medicinal Plants, Faculty of Pharmacy, Future University in Egypt, Cairo 11835, Egypt; 2 Department of Pharmacognosy, Faculty of Pharmacy, Ain-Shams University, Cairo 11566, Egypt; haidygad@pharma.asu.edu.eg; 3 Department of Pharmacognosy, College of Pharmacy, King Saud University, Riyadh 11495, Saudi Arabia; nalmusayeib@ksu.edu.sa; 4 Arab Company for Pharmaceuticals and Medicinal Plants, (Mepaco-Medifood), El-Sharqiya 11361, Egypt; mbishr_2000@yahoo.com

**Keywords:** Brassicaceae, HPLC, GC–MS, glucosinolates, volatile constituents

## Abstract

Brassicaceae comprises various species representing an economically important source of industrial or pharmaceutical crops. The present study aimed to identify glucosinolates (GSLs) and volatile compounds in six Brassicaceae seeds cultivated in Egypt. An (High Performance Liquid Chromatography-Photodiode Array) HPLC–PDA analysis of GSLs in the alcoholic extracts of *Raphanus raphanistrum* L. (Rr), *Raphanus sativus* L. (Rs), *Brassica oleracea* var. *capitata* L. (Boc), *Brassica oleracea* var. *botrytis* L. (Bob), *Brassica rapa* L. (Br), and *Eruca sativa* L. (Es) was carried out using a mixture of 23 standard GSLs. Nineteen GSLs were detected in the studied seeds. Rs had the highest GSL content (135.66 μmol/g Dry weight, DW), while Boc had the lowest GSL content (93.66 μmol/g DW). Glucobrassicin was the major identified compound in Rr, Rs, and Bob. Its highest content was in Rs (28.96 μmol/g DW). Sinigrin was the major identified GSL in Boc (18.02 μmol/g DW), although present with higher content in Bob (22.02 μmol/g DW). Neoglucobrassicin was the major GSL in Br (30.98 μmol/g DW), while glucoerucin was the major GSL in Es (17.84 μmol/g DW). The yields of the steam-distilled oils of the studied seeds ranged between 3.25 ± 0.36 and 9.68 ± 0.25% *v*/*w*. A GC–MS analysis of the oils could detect 3, 23, 18, 16, 7, and 9 compounds in Rr, Rs, Boc, Bob, Br, and Es oils, respectively. Sulfur and nitrogenous compounds predominated in all studied oils except Rs, which contained a higher percentage of alkanes. The major identified compound in Rr oil was 4-isothiocyanato-1-(methylthio)-1-butene (94.77 ± 1.25%), while in Br it was 3-butenyl isothiocyanate (69.55 ± 1.02%), thiolane in Rs (15.15 ± 0.22%), and erucin in Es (97.02 ± 1.514%). Both Boc and Bob had the same major compound 4-(methylthio) butanenitrile, which represented 40.35 ± 1.15 and 50.52 ± 1.02% in both oils, respectively. Radical scavenging activity for both GSL extracts and essential oils on DPPH radical ranged between 18.01 ± 0.72 and 114.28 ± 1.15 µg/mL (IC_50_). The highest antioxidant capacity was for Es oil, while the lowest one was for Rr oil. Generally, it was observed that the GSLs had better antioxidant activity than their corresponding essential oils except for Es oil, which had higher activity. A principal component analysis (PCA) was successfully applied to discriminate among six Brassicaceae seeds based on both HPLC and GC–MS, where complete segregation was achieved among all samples with high correlation between Boc and Bob. Partial Least Squares-Regression (PLS-R) models showed that there is a better correlation between the antioxidant activity and glucosinolate profile when being compared to that of a volatile one. This profiling and variation of GSLs and volatile metabolites of the studied Brassicaceae seeds may be employed in further studies regarding their health-promoting properties.

## 1. Introduction

Brassicaceae (often called Cruciferae), the mustard family, is by far the largest family in Brassicales, having around 338 genera and 3710 species widespread throughout the world [1]. Family members have economic and agricultural values and have been used for culinary or medicinal purposes in several countries for ages. The family includes many common vegetable plants such as broccoli, cabbage, cauliflower, turnips, and radishes. Different parts of the plants are consumed, either leaves, inflorescence, seeds, roots, or stems. They are either eaten as fresh or preserved vegetables, vegetable oils, spices, and condiments. Recently, several studies have shown evidence of the preventive role of the Brassicaceae species against various diseases [2]. Brassicaceae members are the most available dietary source for the valuable glucosinolates (GSLs). Moreover, they are rich in several other classes of secondary metabolites such as polyphenols, vitamins, and minerals [3]. Brassicaceae seeds have been found to contain considerable amounts of glucosinolates, usually higher than other plant tissues [4]. Several known GSLs have been isolated in pure form from Brassicaceae seeds, such as sinigrin from *Brassica nigra*, glucotrapeolin from *Lepidium sativum* [5], glucotropaeolin from *Carica papaya* [6], and gluconasturtiin from *Barbarea verna* [7]. Cleavage of GSLs results in a range of products that may be beneficial or even harmful. Moreover, the nature of their side chains highly affects their in vivo activities, especially indolyl GSLs such as glucobrassicin and neoglucobrassicin, which are widely distributed in the Brassicaceae family [8]. Specific studies have been performed on their anticancer effects due to their glucosinolate (GSL) content or their degradation products, especially isothiocyanates, either through epidemiological or clinical trials with the elaboration of the possible mechanistic pathways [9]. The family species are also known to have antioxidant, antibacterial, and anti-inflammatory activities in addition to their nutritive value [10].

Studying the chemical profile of volatile constituents and GSLs present in different Brassicaceae species is always of interest to process further research on their biochemical and physiological degradation to assess their potential therapeutic or toxic properties. It was found that phytochemical and biological studies on Brassicaceae seeds are scarce relative to other plant parts. Thus, the present work aims to investigate the chemical composition as well as the radical scavenging activity of the essential oils and the GSLs of six Brassicaceae seeds cultivated in Egypt, namely, *Raphanus raphanistrum* L. (Rr, White Radish), *Raphanus sativus* L. (Rs, Red Radish), *Brassica oleracea* var. *capitata* L. (Boc, Cabbage), *Brassica oleracea* var. *botrytis* L. (Boc, Cauliflower), *Brassica rapa* L. (Br, Turnip), and *Eruca sativa* L. (Es, Arugula).

## 2. Results

### 2.1. Identification and Quantification of Glucosinolates Using HPLC

**Figure 1:**
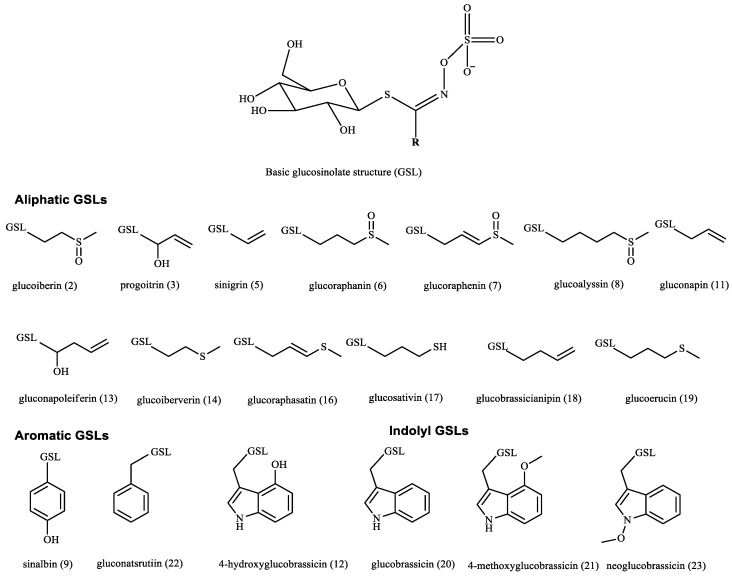
Glucosinolates detected in the alcoholic extracts of the studied Brassicaceae seeds by HPLC–PDA.

**Figure 2:**
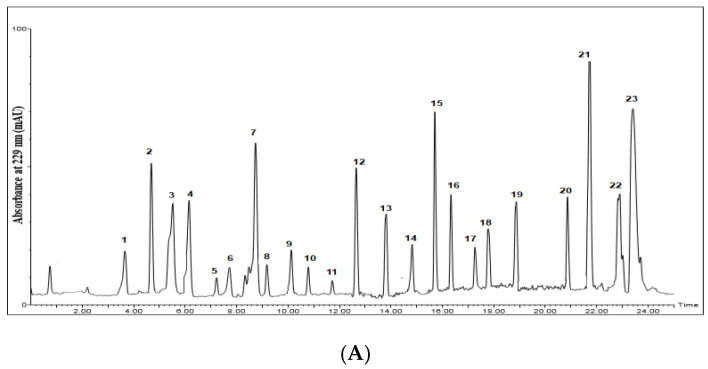

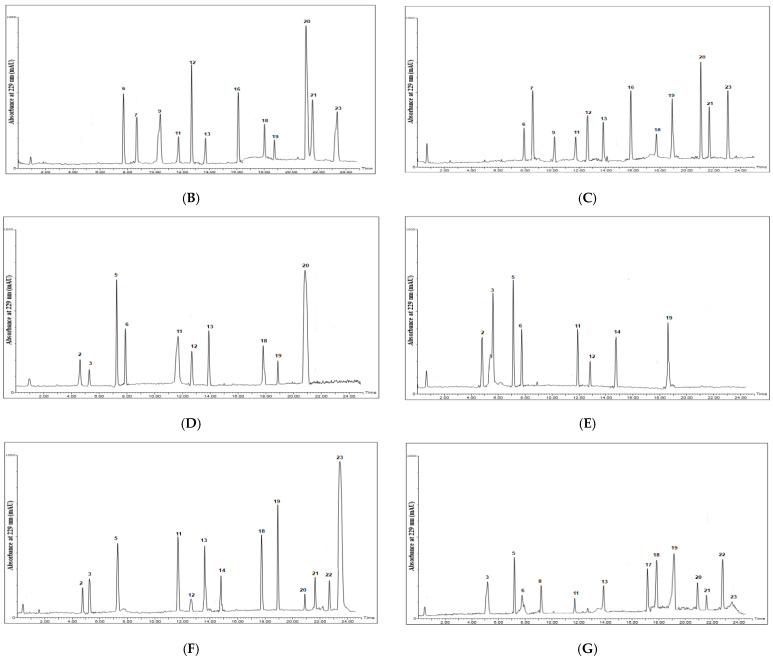
Chromatograms of desulfoglucosinolates in the standard glucosinolate mixture (**A**) and the alcoholic extracts of the tested seeds. (**B**) *Raphanus raphanistrum* L. (**C**) *Raphanus sativus* L. (**D**) *Brassica oleracea* var. *botrytis* L. (**E**) *Brassica oleracea* var. *capitata* L. (**F**) *Brassica rapa* L. (**G**) *Eruca sativa* L. Numbers on charts indicate names of corresponding glucosinolates, which are given.

Quantitative and qualitative identification of GSL content of the desulfated methanol extracts of the seeds was carried out using standard curves created with standard GSLs (Table 1, Figure 1 and Figure 2). Total GSL content varied from 93.66 to 135.66 μmol/g DW in the studied seeds. Rs had the highest GSL content, while Boc had the lowest GSL content. Aliphatic GSL prevailed in Bob (96.02 μmol/g DW), while aromatic GSL had almost the same content in both Rs and Es (≈10 μmol/g DW). Boc and Bob were completely devoid of any aromatic GSL. Rs had the highest content of indolyl GSL (68.59 μmol/g DW), while Boc had the least content of indolyl GSL (6.32 μmol/g DW). Twelve compounds were identified in Rr and Rs with higher GSL content present in Rs (135.77 μmol/g DW). Thirteen compounds were identified in Br and Es, although these 13 compounds were not the same in those species. Ten and eight compounds were identified in both Bob and Boc, respectively. Glucobrassicin was the major identified compound in Rr, Rs, and Bob. Its highest content was in Rs (28.96 μmol/g DW). Sinigrin was the major identified GSL in Boc (18.02 μmol/g DW), although present with higher content in Bob (22.02 μmol/g DW). Neoglucobrassicin was the major GSL in Br (30.98 μmol/g DW), while glucoerucin was the major GSL in Es (17.84 μmol/g DW).

### 2.2. Essential Oils Yield and Composition

**Figure 3:**
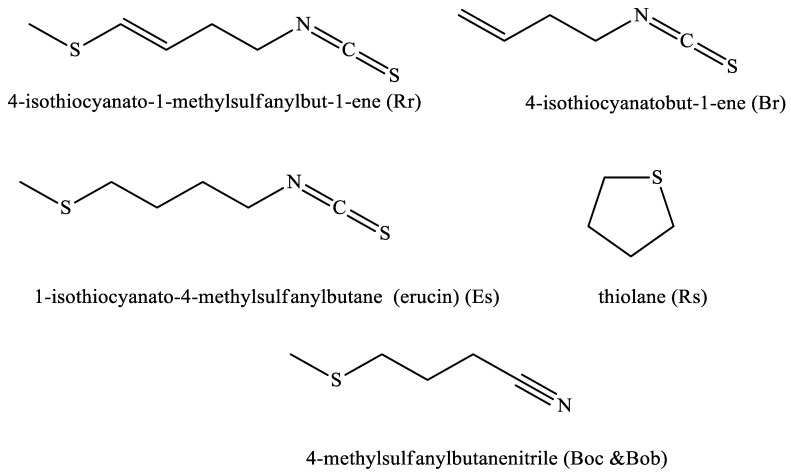
Major compound identified by GC/MS in the volatile oil of the studied seeds. (Rr: *Raphanus raphanistrum,* Rs: *Raphanus sativus*, Boc: *Brassica oleracea* var. *capitatus*, Bob: *Brassica oleracea* var. *botrytis*, Br: *Brassica rapa*, and Es: *Eruca sativa*).

The yield of the steam-distilled oil of the six Brassicaceae seeds ranged between 3.25 ± 0.36 and 9.68 ± 0.25% *v*/*w*. Br oil was completely colorless, and Es was yellowish, while Rr and Rs had a pale yellow color. Both Bob and Boc had a dark yellow color. (Table 2). Es had the lowest yield, while Rr had the highest yield. All oils had a strong characteristic pungent odor. A GC/MS analysis of the oils could detect 3, 23, 18, 16, 7, and 9 compounds in Rr, Rs, Boc, Bob, Br, and Es oils, respectively. A total of 40 compounds were identified in the six oils. Sulfur and nitrogenous compounds predominated in all the studied oils except Rs, which contained a higher percentage of alkanes, although tetrahydrothiophene (thiolane) was the major identified compound in this oil (15.5 ± 0.22%). Moreover, this compound appeared with varying percentages in all tested oils except Rr. The major identified compound in Rr oil was 4-isothiocyanato-1-(methylthio)-1-butene (94.77 ± 1.25%), while in Br it was 3-butenyl isothiocyanate (69.55 ± 1.02%), and in Es it was 4-methylthiobutyl isothiocyanate (also known as erucin) (97.02 ± 1.514%). Both Boc and Bob had the same major compound 4-(methylthio) butanenitrile, which represented 40.35 ± 1.15 and 50.52 ± 1.02% in both oils, respectively. Both Rr and Es oils contained only alkanes and nitrogenous and sulfur compounds. Only one aldehyde, 3-hydroxy-2,2-dimethylpropanal, appeared in Bob oil with a low percentage (0.45 ± 0.02%) (Table 3, Figure 3).

### 2.3. Evaluation of DPPH Radical Scavenging Activity

The radical scavenging activity for both GSL extracts and essential oils on DPPH radical ranged between 114.28 ± 1.15 and 18.01 ± 0.72 µg/mL (IC_50_). The highest antioxidant capacity was for Es oil, while the lowest one was for Rr oil. Generally, it was observed that the GSLs had better antioxidant activity than their corresponding essential oils except for Es oil, which had higher activity (Table 4).

### 2.4. Multivariate Analysis

#### 2.4.1. Principal Component Analysis (PCA)

**Figure 4:**
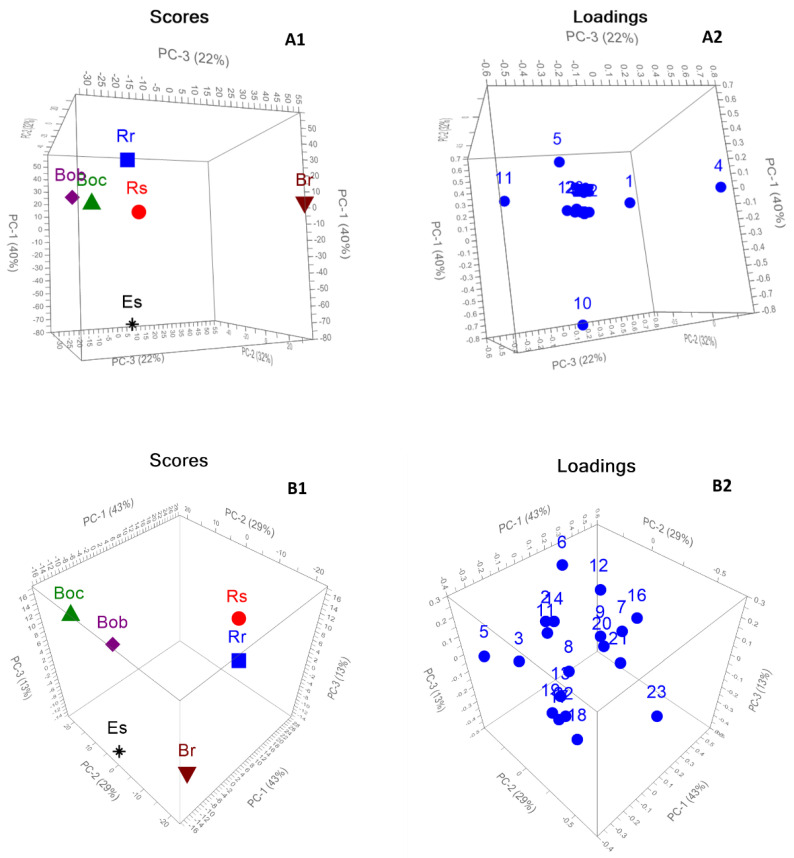
3D PCA score (**A1**) and loading (**A2**) plots based on volatile compounds chemical profiling identified by GC-MS of six Brassicaceae seeds. 3D PCA score (**B1**) and loading (**B2**) plots based on glucosinolates chemical profiling identified by HPLC of six Brassicaceae seeds.

Both HPLC and GC–MS chemical profiling of the six Brassicaceae seeds were assessed using PCA to evaluate the potential importance of both volatile compounds and glucosinolates in the segregation of the collected seeds and the interpretation of the markers responsible for this segregation. Figure 4(A1,A2) displayed the 3D PCA score and loading plots based on GC–MS chemical profiling of the six Brassicaceae seeds. The first 3PCs explained 94% of the total data variance (PC1 40%, PC2 32%, and PC3 22%). The results reveal a significant variance among six seeds based on their volatile compounds as all samples were fully grouped away from each other. Both Br and Boc and Bob were dispersed along positive PC1 completely away from each other. However, it was observed that both Boc and Bob were closely related. This closeness is attributed to the presence of 4-(methylthio)butanenitrile (compound no. **11** as in Table 2) as a distinctive marker, as shown in the loading plot (Figure 4B). Es was clearly described along PC3, totally segregated from other samples. The loading plot revealed that the main descriptive markers accounting for the clear discrimination of Br, Es, and Rr were 3-butenyl isothiocyanate, 4-methylthiobutyl isothiocyanate, and 4-isothiocyanato-1-(methylthio)-1-butene (compound nos. **4**, **10** and **5** as in Table 2), respectively. The plot presented the fall of Rs in the distance close to that of Rr and Boc and Bob.

**Figure 5:**
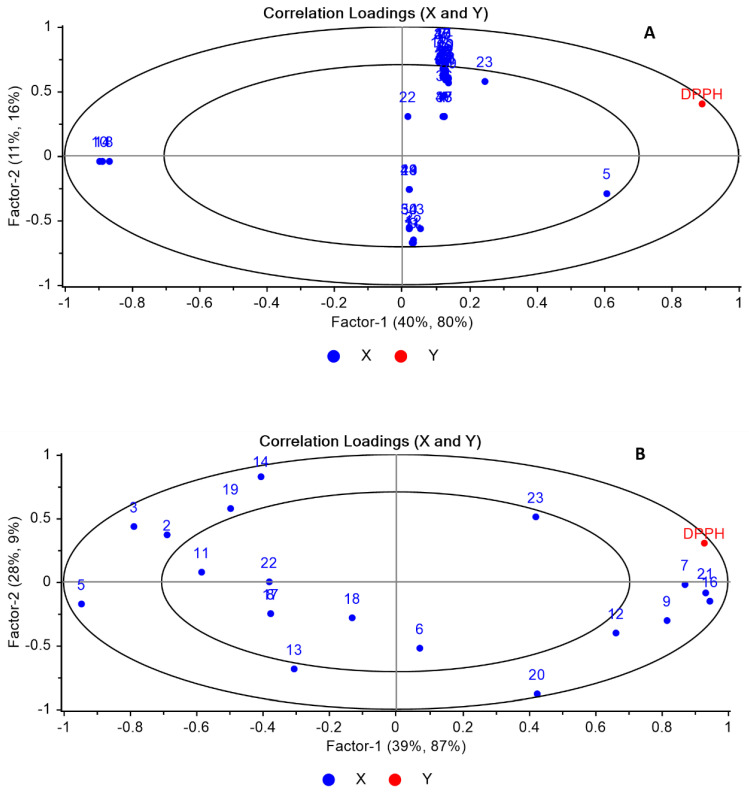
Correlation loading plot (**A**) PLS-R1 for GC-MS data (X matrix) and antioxidant activity (Y matrix), (**B**) PLS-R2 for HPLC data (X matrix) and antioxidant activity (Y matrix). Please refer to Table 1 and Table 3 for compound numbers.

Figure 4(B1,B2) displayed the 3D PCA score and loading plots based on HPLC chemical profiling of the glucosinolates in the six Brassicaceae seeds. The first 3PCs clarified 85% of the data discrepancy (PC1 43%, PC2 29%, and PC3 13%). The PCA score plot based on HPLC chemical profiling was closely associated with that of GC–MS, where a significant discrepancy was observed among six seeds in a similar pattern to that of GC–MS. All samples were completely separated away from each other. The plot revealed that Boc and Bob were still closely related with slight separation. Although sinigrin exhibited high concentration in both Boc and Bob, the loading plot (Figure 5B) revealed that sinigrin and progoitrin (compound nos. **5** and **3** as in Table 3) were marker compounds that caused their separation, respectively. Neoglucobrassicin and glucoraphasatin (compound nos. **23** and **16** as in Table 2) were the segregating markers for Rr and Rs, respectively. Br and Es were located on the opposite side of Rr and Rs with glucobrassicanapin and glucoerucin (compound nos. **18** and **19** as in Table 2) contributing to their segregation.

#### 2.4.2. Partial Least Squares Regression (PLS)

Partial least squares regression (PLS-R) analysis was conducted to establish a correlation between both the volatile compounds and their antioxidant activities. PLS-R1 and PLS-R2 models were constricted by the data matrix X containing the peak area of the GC/MS and HPLC, respectively, and the response vector Y containing the antioxidant activity data.

As shown in Figure 5A, 51% of the X variables in the PLS-R1 model accounted for 96% of the Y variables. However, regarding the PLS-R2 model, as shown in Figure 5B, 67% of the X variables in the PLS-R2 model accounted for 96% of the Y variables.

The ideal number of latent variables (LVs) in the PLS model was calculated using the minimum root mean square error (RMSE) values obtained by cross-validation (CV). The observed fit and the predictive ability of the developed PLS model were evaluated using the following parameters: (1) correlation coefficient R^2^, which measures the correlation between the spectral data and the reference data; (2) the root mean square error of prediction (RMSEP), which indicates the average difference between predicted and measured response values; and (3) the root mean square error of calibration (RMSEC), which shows how reliable the predicted value is in comparison with the reference value at the calibration stage. The low differences between RMSEC and RMSEP reveal the robustness of the model. The ideal PLS models have the lowest value of both RMSEC and RMSEP, with a correlation coefficient R^2^ > 0.99, a slope closer to 1, and an intercept value closer to zero [11]. The PLS-R model parameters, including slope, offset, RMSEC, RMSEV, and R^2^ are shown in Table 5, indicating a moderate prediction ability of the PLS regression model. The PLS-R models showed good linearity and accuracy with R^2^ > 0.98, a slope close to 1 (a value close to 1 means the predicted values are close to the reference), with low differences between RMSEC, and the root mean square error of prediction (RMSEP) reveals the robustness of the model. The prediction performance for the developed models is shown in Table 6.

## 3. Discussion

Brassicaceae members have always been considered one of the most important sources of edible vegetables in several parts of the world. Glucosinolates are mainly concentrated in the seeds of Brassicaceae plants and are also distributed in other vegetative tissues [12]. An HPLC analysis of the alcoholic extracts of the studied seeds revealed that glucobrassicin was the major identified GSL in Rr, Rs, and Bob with the highest content present in Rs (28.96 μmol/g DW). Glucobrassicin is a 3-indolylmethyl GSL prevailing in the members of the Brassicaceae family. This GSL was found to be major in wild radish collected from different parts of the United States but in other tissues than the seeds [13]. Glucobrassicin is a popular indolyl GSL first isolated from *Brassica* and *Raphanus* genera [14]. Its first isolated derivative was neoglucobrassicin (1-Methoxyglucobrassicin), which was identified as major GSL in Br (30.98 μmol/g DW) and detected in less amounts in Rr, Rs, and Es. Other derivatives include 4-methoxyglucobrassicin present in Rr, Rs, Br, and Es and 4-hydroxyglucobrassicin, which was detected in all the studied seeds except Es. Generally, upon chemical or enzymatic hydrolysis of indolyl GSL, a range of involatile indole compounds is produced, exhibiting promising antioxidant and anticarcinogenic activities [15,16]. Glucobrassicin and its hydrolysis product indole-3-carbinol have shown antimicrobial activity against Gram-positive bacteria such as *Enterococcus faecalis*, *Staphylococcus aureus,* and *Staph. saprophyticus* [17]. Sinigrin, which was identified as major GSL in Boc, is an aliphatic GSL widespread in members of the Brassicaceae family. It has a plethora of biological activities such as anti-inflammatory, antioxidant, antimicrobial, and anticancer and wound-healing properties and biofumigation [18]. In wild cabbage collected in England, sinigrin also was the prevailing GSL as well as progoitrin [19]. In the alcoholic extract of Es, glucoerucin was detected as a major GSL, and its hydrolytic volatile product, erucin, was also detected as major in the oil. It has been previously mentioned that both compounds showed anticancer activities. However, glucoerucin in particular has also proven to be the reason why Es meals can be recommended as a nutraceutical tool to relieve pain in diabetic neuropathy patients through H_2_S scavenging and selectively blocking Kv7 potassium channels [20]. Sprouts from the Italian wild *E. sativa* L. had a high percentage of glucosativin. However, sprouts from Central and Eastern European and Tunisian wild seeds had also glucoerucin as the major GSL [21]. Es oil and GSL extract had the highest radical scavenging activity in all tested seed extracts. This may due be to the presence of glucoerucin and erucin present as major in both GSL extract and oil, respectively. Studies show that glucoerucin exhibited antioxidant activity through the induction of phase II enzymes as well as scavenging alkyl hydroperoxides and hydrogen peroxide gathered in cells and peripheral blood and by serving as a precursor of sulforaphane, which is a powerful inducer of detoxifying enzymes [22].

Brassicaceae seed oils have been employed in different industrial and pharmaceutical uses. This work studied the chemical composition of six Brassicaceae seed oils using GC–MS. In Rr oil, only three compounds were identified, representing 99.14% of the total oil. Its major compound, 4-isothiocyanato-1-(methylthio)-1-butene, represented 94.77 ± 1.25%. It was also found to be major in other Rr oil samples collected from China, India, and Japan [23,24,25]. This compound is the corresponding isothiocyanate of glucoraphasatin GSL, which was identified as one of the major compounds (15.21 μmol/g DW) detected in the alcoholic extract of the studied seed. Both have proven to be potential chemoprotective agents through upregulating enzymes involved in detoxifying carcinogens [26]. Rs had tetrahydrothiophene (thiolane) as a major compound in its oil. This sulfur compound was also major in Rs oils from seeds, leaves, and roots from Egypt and China [27,28,29]. Thiolane has been used mainly as an insecticide and herbicide [30]. The major identified compound in Br oil was 3-butenyl isothiocyanate (69.55 ± 1.02%). It is the hydrolytic product of the GSL gluconapin (which was identified in a relatively high amount in the alcoholic extract of the seed; 13.21 μmol/g DW). This compound was also identified in several *Brassica* species oils, such as *B. juncea* and *B. campestris* [31,32]. It proved to be a cytotoxic potential against several cancer cell lines such as prostate carcinoma cells by cell death induction through the inhibition of mitosis and the induction of apoptosis [31]. Both Boc and Bob had the same major compound 4-(methylthio) butanenitrile, which represented 40.35 ± 1.15 and 50.52 ± 1.02%, respectively. This major compound was also detected in the extracts of other *Brassica oleracea* varieties cultivated in other countries such as France, Japan, and the UK [33,34,35]. 4-(methylthio) butanenitrile, also known as iberverin nitrile, is the corresponding isothiocyanate of the GSL glucoiberverin. Erucin (4-methylthiobutyl isothiocyanate) was the major identified compound in Es oil. Several studies have reported this compound to be major in Es seeds or leaves [36,37]. Erucin results from the enzymatic hydrolysis of glucoerucin (which has also been identified in this study as the major GSL in the alcoholic extract of Es seeds). Erucin was first isolated in the 1970s from Es seeds [38]. It could be synthesized from 3-butenyl isothiocyanate (which was identified in the studied oil in a low percentage, 0.47%) in a one-step reaction [39]. Several studies have shown that erucin is a highly promising chemoprotective agent, which could selectively inhibit the growth of several cancer cells such as human pulmonary, ovarian, and hepatoma cells [40,41,42,43].

Generally, it was found that in plants rich in GSLs, the myrosinase enzyme is usually present in the same tissues. When the plant tissue is disrupted, during processing or extraction, myrosinase comes in contact with different GSLs present in the tissues, which may result in their breakdown into the corresponding isothiocyanates and nitriles. However, this breakdown doesn’t usually occur as it depends on storage conditions, temperature, presence of specific proteins, and several other factors [44].

It was observed that all essential oils and GSL extracts of the studied Brassicaceae seeds exhibited good antioxidant activity. It was found that several isothiocyanates and indole derivatives of GLSs are considered as potent inducers of phase II enzymes [45]. Moreover, genes encoding for phase II enzymes contain antioxidant response element domains to which isothiocyanates bind, thus, increasing their expression [46].

A PCA score plot based on both GC–MS and HPLC revealed clear discrimination among six Brassicaceae seeds. The PCA pattern based on the volatile components relayed mainly on the major identified constituents. On the contrary, PCA based on HPLC revealed that the complete chemical profile is necessary to discriminate six seeds, not solely the major components.

By applying the PLS model, the PLS-R1 model revealed that DPPH did not exhibit a positive correlation with any volatile compound, though, compounds nos. **8**, **10,** and **14** are negatively correlated with DPPH as shown in Figure 5A. This result displayed that the antioxidant activity is mainly attributed to all the volatile compounds not just a single marker compound. On the contrary, the results of PLS-R2 showed that glucoraphenin, glucoraphasatin, and 4-methoxyglucobrassicin (compound nos. (**7**, **16**, and **21**)) were located close to each other and positively correlated with DPPH. This result explains the higher antioxidant activities of both Rr and Rs samples as they contained the highest concentrations of compound nos. **7**, **16**, and **21** in comparison to other samples. Nevertheless, singrin (compound no. (5)) is negatively correlated with DPPH, and this highlights the lowest antioxidant activities of Boc, Bob, and ES samples as they displayed the maximum amounts. Variables that are very close to the center are not well described by factor 1 and factor 2.

As shown in Table 5, it was observed that R^2^ > 0.98 indicatinga good correlation between both volatile compounds and the glucosinolates and their antioxidant activity. The results displayed in Table 6 show that the antioxidant activity is correctly predicted with ±5% accuracy.

## 4. Materials and Methods

### 4.1. Plant Material

The seeds of six Brassicaceae plants, namely, *Raphanus raphanistrum* var. *sativus* L. (Rr, White Radish), *Raphanus sativus* var. *sativus* L. (Rs, Red Radish). *Brassica oleracea* var. *capitata* L. (Boc, Cabbage), *Brassica oleracea* var. *botrytis* L. (Boc, Cauliflower), *Brassica rapa* var. *rapa* L. (Br, Turnip), and *Eruca sativa* var. *sativa* L. (Es, Arugula) were obtained from the medicinal farm of the Arab Company for Pharmaceuticals and Medicinal Plants (Mepaco Medifood). The plants’ identity was kindly identified by the agricultural engineer, Mrs. Trease Labib, consultant of plant taxonomy, Ministry of Agriculture and the ex-director of AlOrman Botanical Garden, Giza, Egypt and further confirmed by Dr. Mohamed El-Gebaly, Senior Botanist, National Research Centre, Egypt. Voucher specimens were deposited in the pharmacognosy research lab at Future University in Egypt (CR.1-6).

### 4.2. Chemicals and Solvents

All used GSL standards were purchased from PhytoLab GmbH & Co. (Vestenbergsgreuth, Germany) and Cfm Oskar Tropitzsch GmbH. (Marktredwitz, Germany). Standard ascorbic acid and DPPH were obtained from Sigma-Aldrich Ltd., St Lois, MO, USA.

### 4.3. Extraction of GSLs and Desulfation

One hundred mg of each seed was defatted using n-hexane in a Soxhlet apparatus. The dried defatted seed meal (0.1 gm) was mixed with 2 mL of boiling 70% methanol for 20 min to deactivate myrosinase enzyme. After centrifugation at 3000 rpm for 3 min, the extract was left overnight at room temperature. The collected supernatants were then combined, representing crude GSL. One ml of this extract was added to the DEAE-Sephadex A25 column and activated with 0.1 M sodium acetate. Purified aryl sulfate (200 µL) was added to the column and left overnight to obtain desulfo-GSL, which was diluted with distilled water, filtered, and used for HPLC analysis [47].

### 4.4. Identification and Quantification of Glucosinolates Using HPLC

The analysis was carried out using a 1260 HPLC system (Waters Corporation, Milford, MA, USA) coupled with a PDA detector (set at 229 nm). Sample solutions (100 μg/mL each) were prepared using HPLC analytical grade methanol, filtered through a membrane disc filter (PTFE, 0.2 μm), and degassed by sonication before injection. Samples (injection volume, 10 μL) were introduced to a reverse phase C18 column (ACQUITY UPLC-BEH C18, 1.7 µm particle size, and 2.1 × 50 mm column, Waters Co., USA). Gradient elution was performed using two eluents at a flow rate of 0.2 µL/min: eluent A (100% ultrapure water) and eluent B (100% acetonitrile). Elution was performed according to the following gradient: 10% B (0–10 min), 10–30% B (11–20 min), and 1% B (21–30 min). Peaks and relative peak areas were tentatively identified by comparison with authentic GSL standards, which were desulfated using the same previously mentioned procedure. Serial concentrations of each standard GSL (10–20 µL/mL were used to establish the standard calibration curve. The amount of desulfo-GSL was expressed as µmol/gm DW (dry weight) [47]. GSL names were given according to the standard recommended nomenclature method [48].

### 4.5. Extraction of the Essential Oil and GC–MS Identification

The hydrodistillation was carried out using Clevenger-type apparatus. Two hundred and fifty grams of dried powdered seed from each plant was hydrodistilled for 4 h. The obtained essential oils were received in diethyl ether, dried over anhydrous sodium sulfate, and kept in sealed amber vials at 4 °C until GC–MS analysis. A Shimadzu (Shimadzu Co., Kyoto, Japan) GCMS-QP2010 SE was used for GC analysis. The GC column was a ZB-5 fused silica capillary column with a (5% phenyl)-polymethylsiloxane stationary phase and a film thickness of 0.25 µm. The oven temperature was set at 40 °C for 2 min and then raised at the rate of 4 °C/min up to 220 °C. The injector and detector temperatures were adjusted at 280 and 300 °C, respectively. Helium was used as a carrier gas with a flow rate of 2 mL/min. The sample volume was 0.1 μL, each injected manually in split mode. EI mode was used for recording the mass spectra. The ionization voltage was 70 eV. The ion source temperature was set at 230 °C. A homologous series of *n*-alkanes (C_5_–C_24_) was employed to calculate the retention indices. Compound identification results were obtained by comparing spectra with Wiley MS libraries and comparing the calculated retention indices with the reported literature [49]. Quantitation of relative percentages of individual components was based on peak areas measurement.

### 4.6. Evaluation of DPPH Radical Scavenging Activity

DPPH free radical (2,2-diphenyl-1-picrylhydrazyl) was used to assess the radical scavenging activity of the seed GSLs and oils according to a published procedure [50] with minor modifications. DPPH stock solution was prepared (8 mg/100 mL in methanol). A control prepared by mixing DPPH stock solution (10 mL) with methanol (10 mL) gave an absorbance at 517 nm (Ac) 0.90 ± 0.02 units. Extracts and oils were prepared in different concentrations (20–400 μg/mL), each mixed with equal volumes of DPPH, and left to stand for 30 min in the dark. Absorbance of each mixture (As) was measured at the same absorbance. Standard ascorbic acid (20–100 μg/mL) was used to obtain the calibration curve. Percentage inhibition was calculated as follows: %I = [(Ac−As)/Ac] × 100. IC_50_ (median inhibitory concentration) values were determined using linear regression analysis of the %I vs. extracts concentration.

### 4.7. Multivariate Analysis

Chemical profiling of both volatile compounds (GC–MS) and glucosinolates (HPLC) of the six Brassicaceae seeds was subjected to multivariate analysis. Principal component analysis (PCA) was applied as an unsupervised pattern to afford an overview of all the sample grouping patterns with each other and to identify markers responsible for this pattern [51]. A quantitative calibration model, partial least squares (PLS), was designed to develop a correlation among the volatile compounds, glucosinolates (GC–MS and HPLC peak areas), (X) matrix, and their antioxidant activity (Y) matrix. In this situation, there was no partition of data into models and test sets as just 18 samples for each model were assessed (small data set). The root mean square error (RMSE) and correlation coefficient assessed the PLS-R model capability (R2). PCA, HCA, and PLS were accomplished using CAMO’s Unscrambler^®^ X 10.4 software (CAMO Software AS, Oslo, Norway).

## 5. Conclusions

Phytochemical and biological studies on Brassicaceae seeds are scarce relative to other plant parts. Thus, the present study detailed the accumulation pattern of GSLs and volatile constituents of the seeds of six Brassicaceae species cultivated in Egypt as well as their antioxidant activity. Collectively, the results highlight the distribution of different types of GSLs in the seeds of the studied species and variation in the total GSL content. Rs had the highest GSL content, while Boc had the lowest GSL content. By applying a multivariate analysis, it was obvious that the PCA models based on both the GC–MS and HPLC data revealed a clear discrimination among six Brassicaceae species. In addition, the construction of the PLS-R models for the first time revealed a good correlation between the antioxidant activity and the whole volatile compounds profile, where the glucosinolate mixture revealed significant markers that are mainly responsible for the antioxidant activity. This profiling and variation of GSL and volatile constituents of the studied Brassicaceae seeds may be employed in further studies concerned with the health-promoting properties of each identified metabolite.

## Figures and Tables

**Table 1:** Glucosinolate profile in the studied Brassicaceae seeds.

	Ret Time	R-Group	Common Name	GSL Content (μmol/g DW) *						Class	Linear Equation	Det. Coeff. r2	LOD mg/L	Range mg/L
				Rr	Rs	Boc	Bob	Br	Es					
1	3.65	methyl	Glucocapparin	ND	ND	ND	ND	ND	ND	Aliphatic	y = 43.343x − 1.254	0.9998	0.27	0.23–60.31
2	4.62	3-(Methylsulfinyl)propyl	Glucoiberin	ND	ND	8.25 ± 0.74	5.13 ± 0.11	4.12 ± 0.21	ND	Aliphatic	y = 80.427x + 0.234	0.9997	0.25	0.85–10.92
3	5.45	2-Hydroxybut-3-enyl	Progoitrin	ND	ND	15.21 ± 0.45	3.34 ± 0.15	6.77 ± 0.42	12.35 ± 0.89	Aliphatic	y = 30.468x + 1.257	0.9999	0.55	0.74–94.83
4	6.23	3-(Methylsulfonyl)propyl	Glucoheirolin	ND	ND	ND	ND	ND	ND	Aliphatic	y = 38.502x − 0.236	0.9996	0.05	1.19–38.21
5	7.24	Prop-2-enyl	Sinigrin	ND	ND	18.02 ± 1.02	22.02 ± 0.98	8.12 ± 0.65	14.22 ± 0.55	Aliphatic	y = 90.766x − 0.475	0.9992	0.21	0.89–54.95
6	7.72	4-(Methylsulfinyl)butyl	Glucoraphanin	7.05 ± 0.53	14.96 ± 0.89	12.32 ± 0.52	16.14 ± 0.23	ND	1.23 ± 0.08	Aliphatic	y = 18.618x − 1.235	0.9992	0.23	0.89–28.68
7	8.79	4-(Methylsulfinyl)but-3-enyl	Glucoraphenin	15.11 ± 0.84	8.32 ± 0.44	ND	ND	ND	ND	Aliphatic	y = 20.730x − 0.254	0.9997	0.19	2.20–35.31
8	9.21	5-(Methylsulfinyl)pentyl	Glucoalyssin	ND	ND	ND	ND	ND	2.95 ± 0.07	Aliphatic	y = 81.427x + 0.514	0.9995	0.18	1.11–35.61
9	10.19	4-Hydroxy benzyl	Sinalbin	3.02 ± 0.06	10.21 ± 0.27	ND	ND	ND	ND	Aromatic	y = 67.591x + 1.235	0.9998	0.19	2.30–36.21
10	10.82	6-(Methylsulfinyl)hexyl	Glucohesperin	ND	ND	ND	ND	ND	ND	Aliphatic	y = 29.598x − 0.369	0.9994	0.22	0.99–23.65
11	11.65	but-3-enyl	Gluconapin	4.15 ± 0.23	5.23 ± 0.33	11.11 ± 0.09	15.02 ± 0.47	13.21 ± 0.77	3.44 ± 0.58	Aliphatic	y = 52.109x − 5.158	0.9996	0.21	0.36–52.15
12	12.53	4-hydroxy-Indol-3-ylmethyl	4-hydroxyglucobrassicin	8.59 ± 0.84	16.52 ± 1.02	6.32 ± 0.18	7.72 ± 0.21	0.79 ± 0.07	ND	Indolyl	y = 53.070x − 0.235	0.9994	0.24	0.36–31.26
13	13.98	2-Hydroxypent-4-enyl	Gluconapoleiferin	6.32 ± 0.74	3.65 ± 0.05	ND	17.98 ± 1.05	7.11 ± 0.68	8.54 ± 0.28	Aliphatic	y = 132.13x − 1.258	0.9993	0.55	0.12–12.67
14	14.85	3-(Methylthio)propyl	Glucoiberverin	ND	ND	9.31 ± 0.54	ND	6.95 ± 0.65	ND	Aliphatic	y = 7.5889x − 6.955	0.9994	0.36	1.23–0.65
15	15.75	Benzyl	Glucotropaeolin	ND	ND	ND	ND	ND	ND	Aromatic	y = 43.343x − 2.095	0.9997	0.11	0.89–12.33
16	16.35	4-(Methylthio)-3-butenyl	Glucoraphasatin	15.21 ± 0.98	15.58 ± 0.54	ND	ND	ND	ND	Aliphatic	y = 31.579x + 0.365	0.9996	0.25	1.99–25.89
17	17.26	4-Mercaptobutyl	Glucosativin	ND	ND	ND	ND	ND	13.55 ± 0.58	Aliphatic	y = 29.598x − 0.215	0.9997	0.12	1.23–35.63
18	17.83	Pent-4-enyl	Glucobrassicanapin	4.21 ± 0.07	7.89 ± 0.12	ND	9.06 ± 0.22	14.12 ± 0.42	15.14 ± 0.87	Aliphatic	y = 7.528x − 5.255	0.9997	0.11	0.06–11.65
19	19.01	4-(Methylsulfanyl)butyl	Glucoerucin	12.54 ± 0.87	1.23 ± 0.03	13.12 ± 0.45	7.33 ± 0.32	16.32 ± 0.11	17.84 ± 1.02	Aliphatic	y = 34.257x − 0.266	0.9996	0.23	0.37–52.23
20	21.11	Indol-3-ylmethyl	Glucobrassicin	15.23 ± 0.86	28.96 ± 1.11	ND	26.12 ± 1.23	1.03 ± 0.05	10.18 ± 0.65	Indolyl	y = 53.040x − 1.525	0.9994	0.22	0.99–22.12
21	21.81	4-Methoxyindol-3-ylmethyl	4-methoxyglucobrassicin	10.14 ± 0.45	12.99 ± 0.85	ND	ND	5.25 ± 0.65	4.62 ± 0.44	Indolyl	y = 132.254x − 6.989	0.9997	0.42	2.21–26.95
22	23.01	Phenethyl	Gluconasturtiin	ND	ND	ND	ND	4.12 ± 0.25	10.45 ± 0.47	Aromatic	y = 7.5847x − 2.066	0.9997	0.33	1.25–33.25
23	23.63	N-Methoxyindol-3-ylmethyl	Neoglucobrassicin	13.36 ± 0.74	10.12 ± 0.25	ND	ND	30.98 ± 1.05	1.12 ± 0.05	Indolyl	y = 43.259x + 0.237	0.9996	0.25	0.89–52.23
Total GSL content (μmol/g DW)				114.93	135.66	93.66	129.86	118.89	115.63					
Total Aliphatic GSL (μmol/g DW)				64.59	56.86	87.34	96.02	76.72	89.26					
Total Aromatic GSL (μmol/g DW)				3.02	10.21	0.00	0.00	4.12	10.45					
Total Indolyl GSL (μmol/g DW)				47.32	68.59	6.32	33.84	38.05	15.92					
No of detected compounds				12	12	8	10	13	13					

Rr: Raphanus raphanistrum, Rs: Raphanus sativus, Boc: Brassica oleracea var. capitatus, Bob: Brassica oleracea var. botrytis, Br: Brassica rapa, and Es: Eruca sativa. * Average of three determinations.

**Table 2:** The yield and color of the volatile oils of Brassicaceae seeds.

	Yield (%*v*/*w*) *	Color
Rr	9.68 ± 0.25	pale yellow
Rs	6.43 ± 0.36	pale yellow
Boc	7.02 ± 0.87	dark yellow
Bob	5.55 ± 0.15	dark yellow
Br	8.11 ± 0.25	colorless
Es	3.25 ± 0.36	yellowish

* Average of three determinations.

**Table 3:** GC/MS analysis of volatile oil of Brassicaceae seeds.

No.	Rt *	Compound	Calculated KI **	Reported KI	Relative Abundance % ***					
					Rr	Rs	Boc	Bob	Br	Es
**Sulfur and Nitrogenous Compounds**										
1	5.200	2-methylbut-3-enenitrile	689	689	2.07 ± 0.82	nd	nd	nd	24.03 ± 0.98	nd
2	7.897	isothiocyanatoethane	887	885	nd	nd	nd	0.66 ± 0.11	nd	nd
3	8.024	3-isothiocyanatoprop-1-ene	846	847	nd	0.90 ± 0.05	2.84 ± 0.32	9.86 ± 0.67	0.84 ± 0.02	nd
4	11.173	4-isothiocyanatobut-1-ene	982	988	nd	nd	2.71 ± 0.12	nd	**69.55 ± 1.02**	0.47 ± 0.06
5	11.795	4-isothiocyanato-1-methylsulfanylbut-1-ene	1438	1441	**94.77 ± 1.25**	nd	nd	5.14 ± 0.25	nd	nd
6	17.681	thiolane	1413	1415	nd	**15.15 ± 0.22**	7.14 ± 0.65	2.33 ± 0.04	0.60 ± 0.05	0.30 ± 0.02
7	17.951	1-(6-methylpyridin-3-yl)ethanone	1157	1162	nd	4.20 ± 0.05	nd	nd	nd	nd
8	18.569	5-(methylsulfanyl)pentanenitrile	1149	1154	nd	nd	nd	nd	nd	1.06 ± 0.32
9	21.463	1-methylpyrrolidin-2-one	1010	1012	nd	nd	4.51 ± 0.25	7.72 ± 0.88	nd	nd
10	25.505	1-isothiocyanato-4-methylsulfanylbutane (erucin)	1168	1172	nd	nd	nd	nd	nd	**97.02 ± 1.51**
11	25.600	4-methylsulfanylbutanenitrile	1050	1051	nd	nd	**40.35 ± 1.15**	**50.52 ± 1.02**	nd	0.40 ± 0.08
12	25.610	2-isothiocyanatoethylbenzene	1461	1465	nd	nd	11.28 ± 0.54	11.38 ± 0.42	nd	nd
					**96.84**	**20.25**	**68.83**	**87.61**	**95.02**	**99.25**
**Alkanes**										
13	4.616	3,5-dimethyloctane	926	926	nd	nd	nd	nd	0.13 ± 0.02	nd
14	4.619	2-methylheptane	765	767	nd	nd	nd	nd	nd	0.07 ± 0.01
15	4.728	2,5-dimethylhexane	733	733	nd	2.73 ± 0.84	nd	0.56 ± 0.5	nd	nd
16	5.350	Octane	800	800	nd	8.83 ± 0.52	2.61 ± 0.78	0.95 ± 0.05	nd	0.09 ± 0.02
17	8.260	Nonane	900	900	nd	1.12 ± 0.10	nd	nd	nd	nd
18	11.388	Decane	997	999	nd	8.18 ± 0.57	nd	1.41 ± 0.12	nd	0.18 ± 0.01
19	11.399	4-methyldodecane	1258	1260	nd	nd	4.38 ± 0.21	nd	nd	nd
20	12.122	4-methyldecane	1060	1062	nd	1.71 ± 0.32	0.85 ± 0.06	nd	nd	nd
21	13.455	Tetradecane	1400	1400	nd	1.96 ± 0.14	0.97 ± 0.08	0.32 ± 0.02	nd	nd
22	14.601	Undecane	1100	1100	nd	nd	nd	nd	2.66 ± 0.15	0.32 ± 0.04
23	14.621	Dodecane	1200	1200	2.30 ± 0.25	14.18 ± 0.77	7.09 ± 0.54	2.38 ± 0.08	nd	nd
24	15.019	2-methyldecane	1155	1160	nd	nd	0.86 ± 0.14	nd	nd	nd
25	16.572	2-methylundecane	1162	1166	nd	2.61 ± 0.12	0.93 ± 0.06	nd	nd	nd
26	18.079	2,6-dimethylundecane	1222	1216	nd	2.67 ± 0.64	0.84 ± 0.08	nd	nd	nd
27	19.517	2-methyldodecane	1268	1265	nd	2.00 ± 0.144	nd	nd	nd	nd
28	20.575	Tridecane	1300	1300	nd	2.80 ± 0.87	1.23 ± 0.02	0.38 ± 0.01	nd	nd
					**2.30**	**48.79**	**19.76**	**6.00**	**2.79**	**0.66**
**Cyclic hydrocarbons**										
29	5.033	1,3-dimethyl- cis-cyclohexane	779	778	nd	2.71 ± 0.11	nd	0.55 ± 0.01	nd	nd
30	5.075	1,4-dimethyl- cis-cyclohexane	774	777	nd	0.67 ± 0.05	nd	nd	nd	nd
31	6.400	ethylcyclohexane	831	831	nd	nd	nd	nd	nd	nd
32	12.463	butylcyclohexane	1028	1030	nd	2.13 ± 0.04	1.05 ± 0.08	nd	nd	nd
33	13.244	cyclododecanol	1571	1575	nd	2.90 ± 0.11	1.49 ± 0.26	nd	nd	nd
34	13.247	11,11-dimethyl-bicyclo[8.2.0]dodecane	1472	1476	nd	nd	nd	0.48 ± 0.04	nd	nd
35	14.998	2-methyldecahydro naphthalene	1159	1161	nd	1.55 ± 0.05	nd	nd	nd	nd
					**0.00**	**9.96**	**2.54**	**1.03**	**0.00**	**0.00**
**Other classes**										
36	4.770	3-methylbutyl 2-methylpropanoate	996	998	nd	0.51 ± 0.03	nd	nd	nd	nd
37	5.846	tetrachloroethene	809	814	nd	7.07 ± 0.74	nd	nd	nd	nd
38	7.676	hepta-1,5-diene	689	691	nd	5.58 ± 0.36	nd	nd	1.04 ± 0.12	nd
39	10.175	nonanal	1105	1108	nd	6.04 ± 0.42	3.71 ± 0.03	nd	nd	nd
40	13.567	3-hydroxy-2,2-dimethylpropanal	864	865	nd	nd	nd	0.45 ± 0.02	nd	nd
					**0.00**	**19.20**	**3.71**	**0.45**	**1.04**	**0.00**
**Total number of identified compounds**					**3**	**23**	**18**	**16**	**7**	**9**
**Total %**					**99.14**	**98.20**	**94.84**	**95.09**	**98.85**	**99.91**

Rr: *Raphanus raphanistrum*, Rs: *Raphanus sativus*, Boc: *Brassica oleracea* var. *capitatus*, Bob: *Brassica oleracea* var. *botrytis*, Br: *Brassica rapa*, Es: *Eruca sativa*, nd = not detected, and * Rt: Retention time, ** KI: Kovats index, *** Average of three analyses.

**Table 4:** DPPH radical scavenging activity of GSLs and essential oil of the studied Brassiccaceae seeds.

Sample	DPPH Radical Scavenging Activity (IC_50_ µg/mL)	
	GSLs	Essential Oils
Rr	90.21 ± 1.59	114.28 ± 1.15
Rs	81.31 ± 1.00	119.86 ± 1.95
Boc	50.79 ± 1.98	67.56 ± 2.43
Bob	32.64 ± 1.87	70.25 ± 0.31
Br	59.81 ± 1.63	86.09 ± 2.60
Es	25.18 ± 1.55	18.01 ± 0.72
Ascorbic acid	5.38 ± 0.56	

The results are presented as the mean of three replicates for the samples ± SD.

**Table 5:** The partial least squares regression model parameters used for prediction.

Antioxidant Activity	Data Type	PLS			
		Slope	Offset	RMSE	R^2^
DPPH (Volatile compounds)	Cal.	0.9851	1.1770	4.1312	0.9851
	Val.	0.9686	1.8793	5.1221	0.9796
DPPH (Glucosinolates)	Cal.	0.9950	0.2920	1.6371	0.9950
	Val.	0.9899	0.5609	2.4295	0.9902

RMSE: Root mean squared error; R^2^: Correlation; Cal.: Calibration; and Val.: Validation.

**Table 6:** Results of calibration and predictive ability of the PLS models.

	DPPH (Volatile Compounds)		DPPH (Glucosinolates)	
	Y Reference	Y Predicted	Y Reference	Y Predicted
Rr1	113.08	116.51	90.19	86.96
Rr2	114.39	116.40	88.63	90.44
Rr3	115.37	116.29	91.80	87.48
Rs1	119.16	117.59	81.92	82.00
Rs2	122.06	117.56	81.85	82.93
Rs3	118.35	117.54	80.16	81.08
Boc1	67.76	72.52	50.73	50.81
Boc2	65.04	73.25	48.85	50.43
Boc3	68.89	73.98	52.80	51.19
Bob1	70.14	63.41	31.91	32.93
Bob2	70.01	62.76	31.25	31.39
Bob3	70.60	64.07	34.77	34.48
Br1	88.49	86.27	59.89	60.71
Br2	86.45	86.05	61.39	60.18
Brs	83.33	86.48	58.14	61.24
Es1	18.29	19.13	25.28	24.43
Es2	17.19	17.77	23.58	22.39
Es3	18.55	20.49	26.67	27.20

## Data Availability

Data are available upon request from the first author.
